# NKGD2 Ligands (NKG2DLs) in Breast Cancer: In Silico Analysis and Narrative Review

**DOI:** 10.3390/ijms27041848

**Published:** 2026-02-14

**Authors:** Jesús Peña-López, Angelo Gámez-Pozo, Lucía Trilla-Fuertes, Fernando Becerril-Gómez, Marta Mendiola, Victoria Heredia, Laura Yébenes, Beatriz Castelo, Virginia Martínez-Marín, Enrique Espinosa, Pilar Zamora, Alfonso Alba-Bernal, Cristina Aguirre-Portolés, Antonio Pérez-Martínez

**Affiliations:** 1 Medical Oncology Department, Hospital Universitario La Paz, Universidad Autónoma, 28046 Madrid, Spain; 2 Molecular Oncology Laboratory, Institute of Medical and Molecular Genetics-INGEMM, IdiPAZ-Instituto de Investigacion Sanitaria del Hospital Universitario La Paz, 28029 Madrid, Spain; 3 CIBERONC-ISCIII (Biomedical Research Networking Center on Oncology—Carlos III Health Institute), 28029 Madrid, Spain; 4 Department of Pathology, Hospital Universitario La Paz, 28046 Madrid, Spain; 5 CIBERER-ISCIII (Biomedical Research Networking Center on Rare Diseases—Carlos III Health Institute), IdiPAZ-CNIO Pediatric Onco-Hematology Clinical Research Unit, IdiPAZ-Instituto de Investigacion Sanitaria del Hospital Universitario La Paz, 28029 Madrid, Spain; 6 Pediatric Hemato-Oncology Department, Hospital Universitario La Paz, Universidad Autónoma, 28046 Madrid, Spain

**Keywords:** breast cancer, NKG2D, NKG2DL, in silico

## Abstract

Breast cancer (BC) is a global health problem. BC is a biologically heterogeneous disease in which novel immunotherapeutic strategies are needed, particularly in the metastatic setting. The NKG2D/NKG2D ligand (NKG2DL) axis is a key component of innate antitumor immunity and represents a potential therapeutic target, but its relevance in BC has not been fully characterized. We performed an in silico analysis of NKG2DL expression in BC cell lines, healthy breast tissue, and tumor samples using publicly available transcriptomic databases (DSMZCellDive, ShinyTHOR, GTEx, TCGA, Human Protein Atlas), complemented by survival analyses from TCGA and KMPlot and a narrative review of the literature. NKG2DL transcripts were consistently expressed in BC cell lines and tumor tissues, with higher expression observed in ductal histology, higher tumor stage, and basal molecular subtype. Survival analyses showed heterogeneous and generally weak associations between individual NKG2DLs and clinical outcomes. In silico proteomics data are scarce, but the narrative review showed that NKG2DLs are expressed by immunohistochemistry in tumor tissues but absent in surrounding healthy tissues. The literature review also revealed concomitant dysfunction of NKG2D+ effector cells due to multiple resistance mechanisms (including ligand shedding). We also review potential therapeutic approaches.

## 1. Introduction

Breast cancer (BC) is a global health issue. In 2022, it was the second most frequently diagnosed cancer, accounting for around 2.3 million new cases or approximately one in nine cancer diagnoses worldwide (11.6% of all cancers). It is also the fourth largest cause of cancer-related mortality, responsible for an estimated 0.6 million deaths in 2022 (6.9% of all deaths from cancer). In women, it is the most common tumor and the leading cause of cancer mortality [1]. BC is a biologically heterogeneous disease, with different molecular subtypes with prognostic and therapeutic implications. The triple-negative subtype (TNBC) is particularly important due to its greater aggressiveness, poorer prognosis, and fewer treatment options [2]. Perou et al. established four molecular intrinsic subtypes based on a 50-gene expression signature (PAM50): Luminal A, Luminal B, HER2-enriched and basal-like [3]. In clinical practice these subtypes are subrogated through immunohistochemistry (IHC) of four key biomarkers: estrogen receptor (ER), progesterone receptor (PR), human epidermal growth factor receptor 2 (HER2/ERBB2) and Ki67 (proliferation marker). Depending on the expression of these proteins, tumors are classified as: luminal or hormone receptor-positive (ER+/PR+), HER2-positive (HER2+) or triple-negative (ER-/PR-/HER2-) [2]. There is approximately 80% concordance between the basal molecular subtype and TNBC surrogate subtype [4].

There have been multiple therapeutic innovations in metastatic BC. Traditionally, there was classic chemotherapy, hormone therapy (for luminal tumors), and anti-HER2 targeted therapy (for HER2-positive tumors). However, new treatments have been introduced in recent years:

- Immune checkpoint inhibitors have been incorporated in triple-negative cases if PD-L1 (CD274) is positive (around 40% of the trial population) [5].
- Antibody–drug conjugates (ADCs). Sacituzumab–govitecan (anti-Trop2/TACSTD2) has demonstrated benefits in TNBC and luminal BC [6,7]. Trastuzumab–deruxtecan (antiHER2) has not only demonstrated benefits in HER2-positive tumors, but also in HER2-low tumors (expression present but insufficient to meet the classic criteria for positivity) [8,9]. Others, such as enfortumab–vedotin (anti-Nectin4), are currently being studied [10].

Nevertheless, metastatic BC is incurable in practically most cases and the aim of systemic cancer-specific treatment is palliative [11]. Overall survival rates in the metastatic setting achieved in the luminal and HER2 subtypes are around 5 years, while in TNBC the survival rate is around 1–2 years [2].

The Natural Killer Group 2 member D (NKG2D) receptor plays an important role in protecting the host against infections and cancer. It is constitutionally expressed in γδ T lymphocytes, CD8+ αβ T lymphocytes, and NK cells. It modulates the activation of these lymphocytes by recognizing ligands on target cells such as major histocompatibility complex class I-related proteins A and B (MICA and MICB) and UL16 binding proteins 1–6 (ULBP1, ULBP2, ULBP3, ULBP4/RAET1E, ULBP5/RAET1G and ULBP6/RAET1L). The expression of these ligands can be induced in cells that are infected, damaged by DNA, undergoing carcinogenesis, or stressed by other inducers. Thus, cellular immunity is promoted to eliminate cells that express the ligand [12,13]. These ligands are not widely expressed on healthy adult tissue so NKG2D ligands may present a useful target for immunotherapeutic approaches in cancer [14].

The NKG2D/NKG2DL axis is being targeted in pediatric leukemia with chimeric antigen receptor (CAR) anti-NKG2DL or with ex vivo IL-15-stimulated NK cells [15,16]. The question arises as to whether it is possible to transfer this approach to BC. Notably, early-phase clinical trials are being conducted with CAR-based adoptive cell therapy against NKG2DLs in solid tumors, including BC [17]. To assess the feasibility of this strategy, there are two aspects to consider. First, it is necessary to determine whether NKG2DL expression exists through in silico analysis in different databases. Second, the available evidence regarding BC (especially TNBC) and NKG2DLs must be analyzed.

## 2. In Silico Analysis

### 2.1. In Silico Analysis of Cell Lines

DSMZCellDive [https://celldive.dsmz.de/ (accessed on 23 December 2025)] contains RNA sequencing (RNA-seq) transcriptome data (expressed as TPM, transcripts per million) of different immortalized human cell lines (breast cancer *n* = 29, leukemia/lymphoma *n* = 101, neuroblastoma *n* = 18 and retinoblastoma *n* = 9) (Table S1) [18]. We focused specifically on NKG2DL genes and breast cancer cell lines (Table S2) [19]. In the case of breast cancer, the values for CD274, TACSTD2, ERBB2, and NECTIN4 (genes currently being targeted) were added.

**Figure 1:**
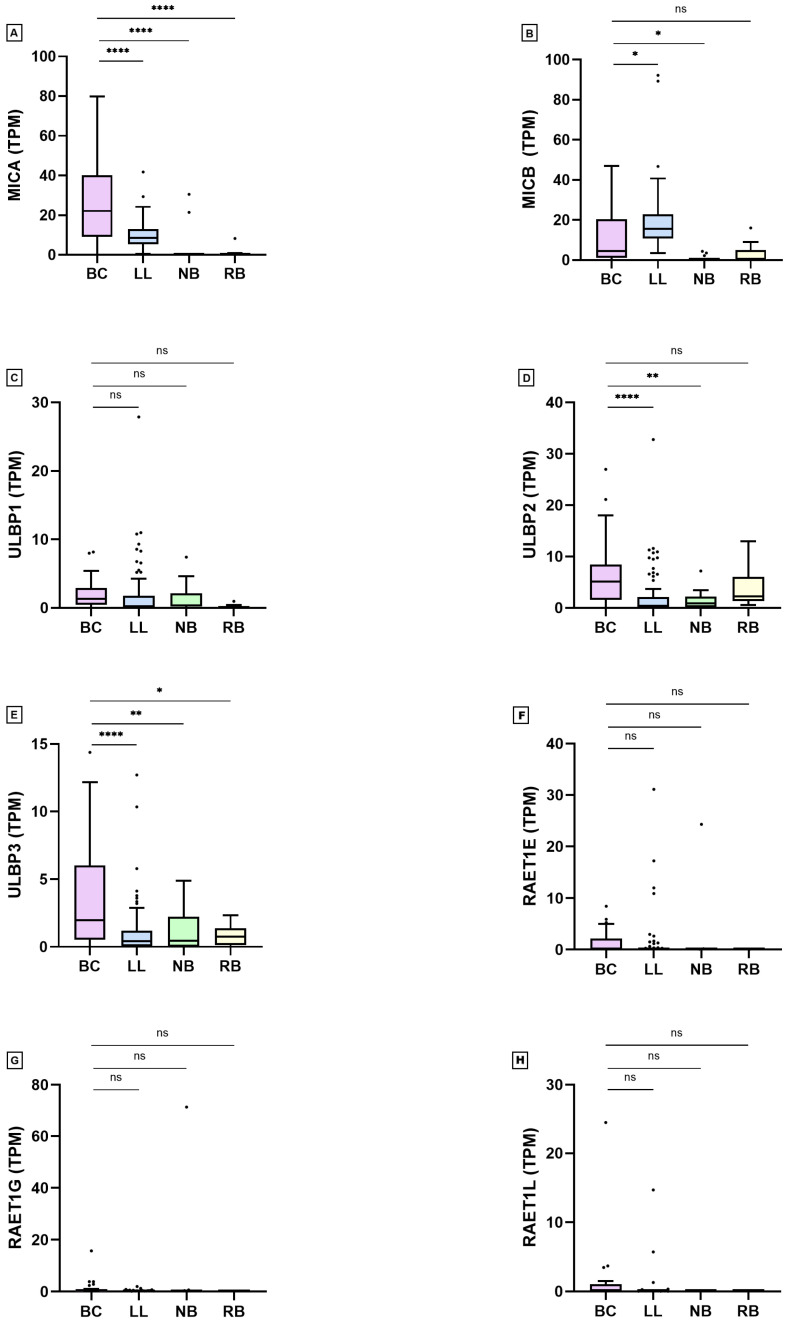
NKG2DL RNA expression in TPM (Tukey boxplot) in different immortalized human cell lines: MICA (**A**), MICB (**B**), ULBP1 (**C**), ULBP2 (**D**), ULBP3 (**E**), RAET1E (**F**), RAET1G (**G**) and RAET1L (**H**). BC = breast cancer (*n* = 29). LL = leukemia/lymphoma (*n* = 101). NB = neuroblastoma (*n* = 18). RB = retinoblastoma (*n* = 9). Horizontal line represents direct comparisons (Student’s *t*-test). ns = non-significant. * = *p* < 0.05. ** = *p* < 0.01. **** = *p* < 0.0001. Statistical analyses were performed and figures were generated using GraphPad Prism for Windows, Version 8.4.2 (GraphPad Software, Boston, MA, USA, http://www.graphpad.com/).

**Figure A1:**
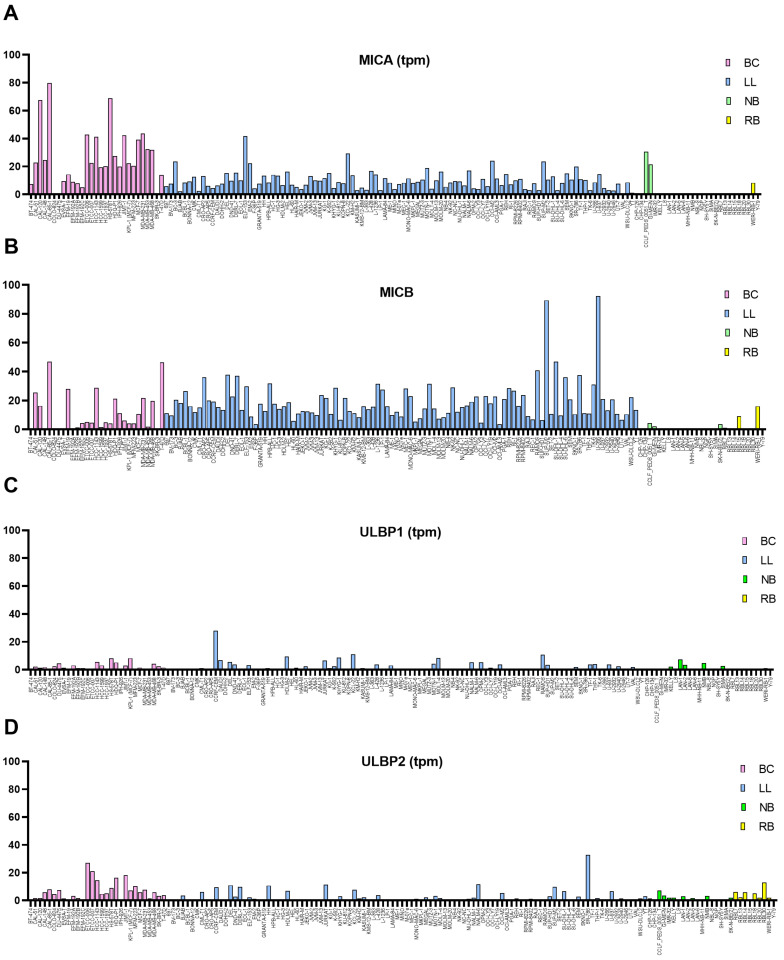
RNA expression in TPM in different immortalized human cell lines of MICA (**A**), MICB (**B**), ULBP1 (**C**) and ULBP2 (**D**). BC = breast cancer (*n* = 29). LL = leukemia/lymphoma (*n* = 101). NB = neuroblastoma (*n* = 18). RB = retinoblastoma (*n* = 9).

**Figure A2:**
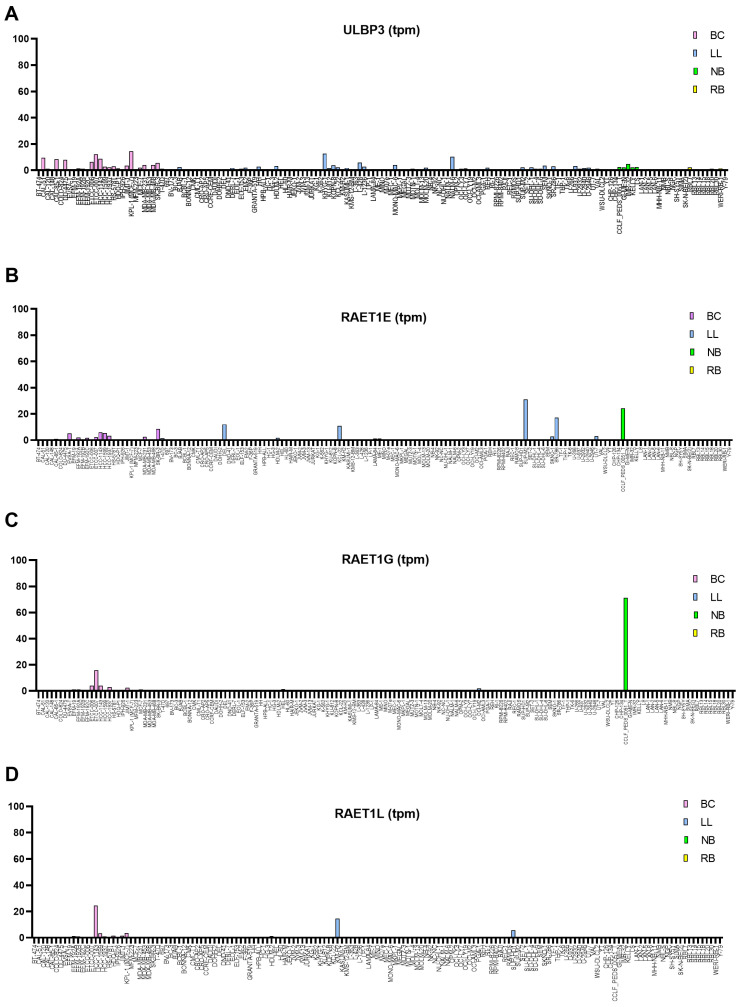
RNA expression in TPM in different immortalized human cell lines of UBLP3 (**A**), RAET1E (**B**), RAET1G (**C**) and RAET1L (**D**). BC = breast cancer (*n* = 29). LL = leukemia/lymphoma (*n* = 101). NB = neuroblastoma (*n* = 18). RB = retinoblastoma (*n* = 9).

The expression of NKG2DLs in the different human cell lines (grouped by tumor) is shown in Figure A1 and Figure A2. The data obtained are illustrated in Table 1 and Figure 1. When specifically analyzing BC cell lines, no statistically significant differences in gene expression were found when stratified by primary vs. metastatic origin or by luminal vs. basal subtype.

**Figure A3:**
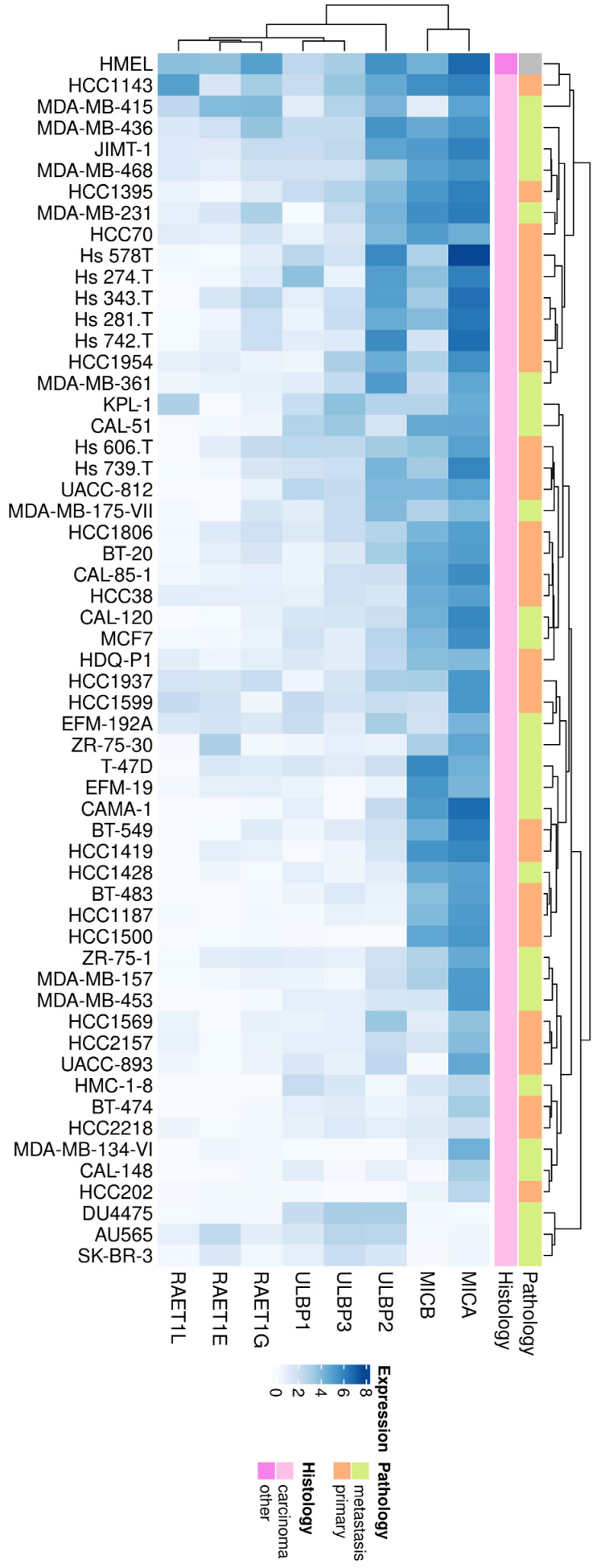
Heatmap of RNA expression of NKG2DL in 60 immortalized human BC cell lines [20]. Obtained from https://alexismurillo.shinyapps.io/ShinyThor/ (accessed on 6 February 2026). Parameters: Multiple Analytes > Choose your module of interest: Gene Expression > Write your analytes of interest (one analyte per line): MICA//MICB//ULBP1//ULBP2//ULBP3//RAET1E//RAET1G//RAET1L > Choose a specific group of cell lines: Breast.

ShinyTHOR [https://alexismurillo.shinyapps.io/ShinyThor/ (accesed on 6 February 2026)] is a web app for access to multi-omic data from different datasets (CCLE, miRTarBase, circInteractome, and the Genomics of Drug Sensitivity in Cancer) [20]. This allowed us to create a heatmap of NKG2DL expression in 60 immortalized human BC cell lines (Figure A3).

### 2.2. In Silico Analysis of Healthy Tissue

The Adult Genotype Tissue Expression (GTEx) Project [http://www.gtexportal.org/home/ (accessed on 23 December 2025)] collected samples from up to 54 non-diseased tissue sites across nearly 1000 deceased individuals [21]. It was combined with RNA-seq transcriptome data of NKG2DL genes (collected as nTPM, normalized transcripts per million) present in The Human Protein Atlas [https://www.proteinatlas.org/ (accessed on 23 December 2025)] [22]. From this, we selected the 514 breast samples (Table S3).

**Figure A4:**
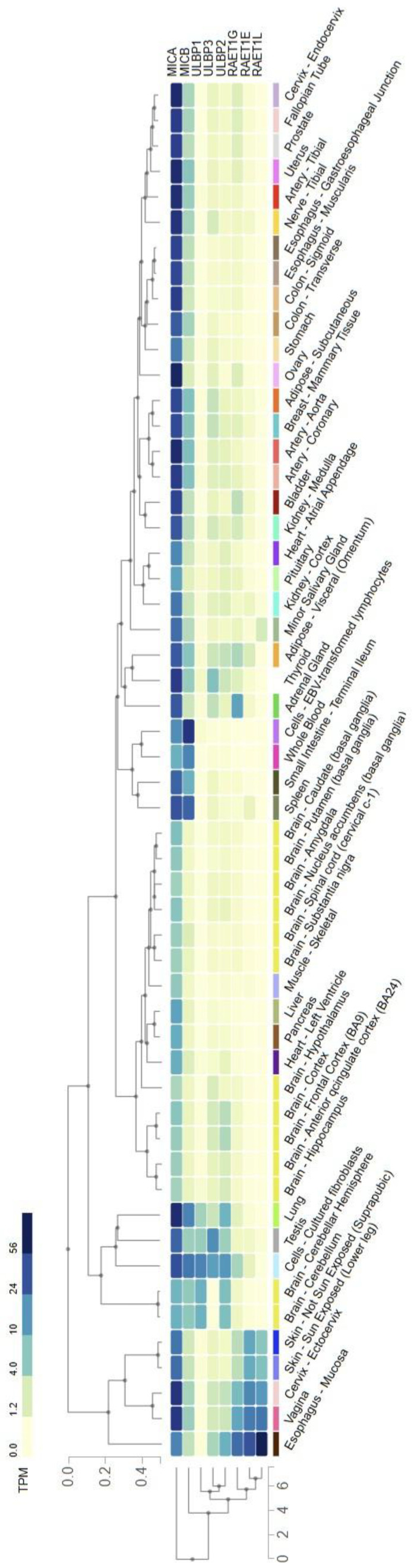
Heatmap of RNA expression in TPM of NKG2DL in different healthy tissues. Obtained from http://www.gtexportal.org/ (accessed on 23 December 2025). Parameters: Expression > Multi-Gene Query > Enter Gencode IDs or Gene Names: MICA//MICB//ULBP1//ULBP2//ULBP3//RAET1E//RAET1G//RAET1L > Select Tissues: All.

The expression of NKG2DLs in healthy breast tissues from the GTex database is illustrated in Table 2. The heatmap of NKG2DL expression in different healthy tissues is shown in Figure A4.

The Human Protein Atlas was also used to evaluate the protein expression of ULBP1, ULBP2, and ULBP3 (the only ones for which information was available). No protein expression was detected in ULBP1 and ULBP2. “Low” protein expression of ULBP3 was detected in the glandular component.

### 2.3. In Silico Analysis of Tumor Tissue

The Cancer Genome Atlas (TCGA) breast cancer database, contained in cBioportal [http://www.cbioportal.org/ (accessed on 23 December 2025)], includes clinical–pathological information from 1084 breast cancer samples [23,24,25,26]. It was combined with RNA-seq transcriptome data of NKG2DL genes (collected as pTPM, protein-transcripts per million) present in The Human Protein Atlas [https://www.proteinatlas.org/ (accessed on 23 December 2025)] [22]. Then, we selected the 1009 samples from patients diagnosed with invasive breast carcinoma (Table S4). In the case of breast cancer, the values for CD274, TACSTD2, ERBB2, and NECTIN4 (genes currently being targeted) were added.

**Figure 2:**
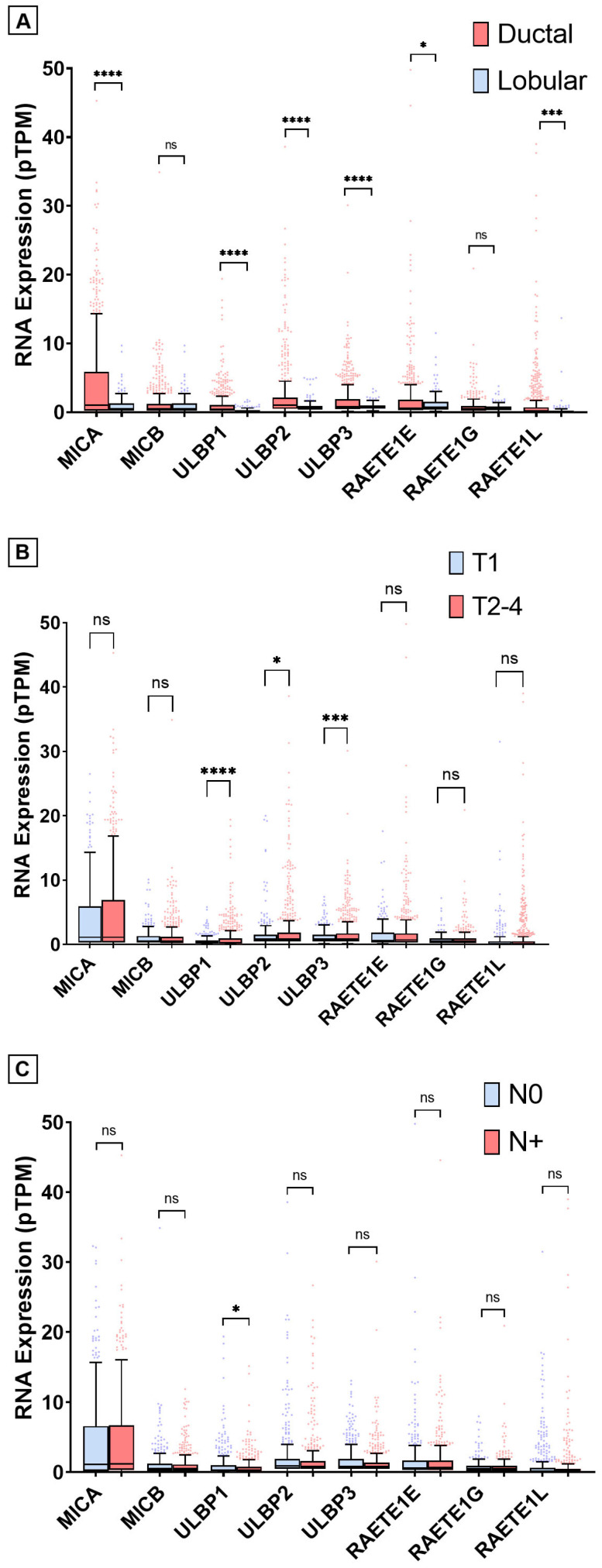
RNA expression in pTPM (Tukey boxplot) by histology (**A**), tumor stage (**B**) and nodal stage (**C**). N zigzag line represents direct comparisons (Student’s *t*-test). ns = non-significant. * = *p* < 0.05. *** = *p* < 0.001. **** = *p* < 0.0001. Statistical analyses were performed and figures were generated using GraphPad Prism for Windows, Version 8.4.2 (GraphPad Software, Boston, MA, USA, http://www.graphpad.com/).

Figure A4, obtained from The Human Protein Atlas, shows the expression of NKG2DLs in different tumors (using TCGA data). The baseline characteristics of the 1009 BC patients are summarized in Table 3. Figure 2 shows the differences in expression by histology (ductal vs. lobular), tumor stage code (T1 vs. T2–4), and nodal stage code (N0 vs. N+). Ductal histology is associated with higher NKG2DL expression (except MICB). T ≥ 2 is associated with higher ULBP1-ULBP2-ULBP3 expression. In N+, higher expression was only detected in ULBP1.

**Figure 3:**
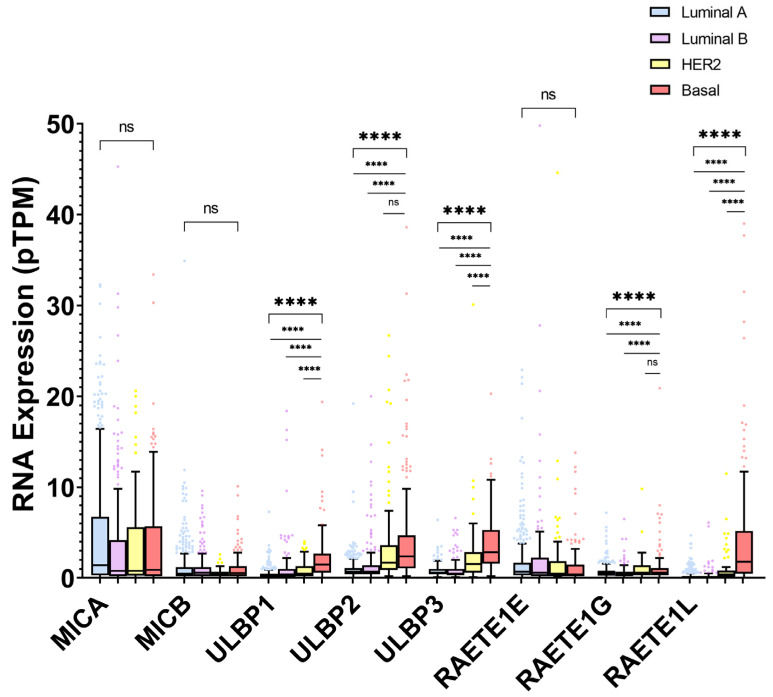
RNA expression in pTPM (Tukey boxplot) by molecular subtype. N zigzag line represents ANOVA test. Horizontal line represents direct comparisons versus basal subtype (Student’s *t*-test). ns = non-significant. **** = *p* < 0.0001. Statistical analyses were performed and figures were generated using GraphPad Prism for Windows, Version 8.4.2 (GraphPad Software, Boston, MA, USA, http://www.graphpad.com/).

Figure 3 shows the differences in expression by molecular subtype. There are significant differences in ULBP1, ULBP2, ULBP3, RAET1G, and RAET1L. In these cases, the basal subtype has higher levels than the other subtypes (significance is not detected only versus HER2 for ULBP2 and RAET1G).

The Human Protein Atlas was also used to evaluate the protein expression of ULBP1, ULBP2, and ULBP3 (the only ones for which information was available). In ULBP1, “medium” protein expression was recorded in 1/11 samples. In ULBP3, “medium” protein expression was recorded in 1/11 samples. In ULBP2, “weak” staining was recorded in <25%.

With the TCGA data, a survival analysis (estimated using the Kaplan–Meier method) could be performed. The median follow-up was 31.4 months (95%CI: 28.6–34.3) and death had occurred in 141 patients (14.0%). The median overall survival (mOS) was 132.0 months (95%CI: 115.3–148.7).

**Figure 4:**
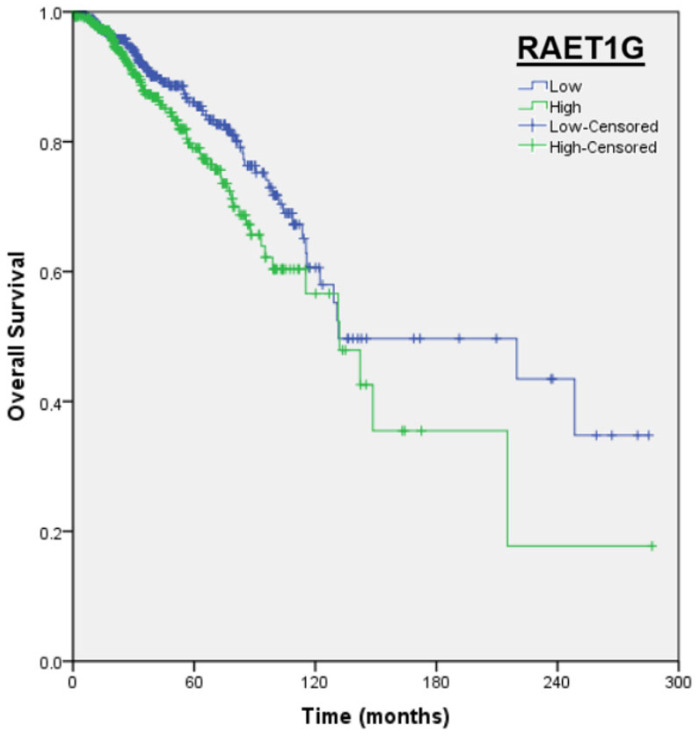
Survival curve for overall survival stratified by low–high levels of RAET1G. Survival analyses were performed using IBM SPSS Statistics for Windows, Version 19.0 (IBM Corporation, Armonk, NY, USA).

A univariate analysis was conducted using the Cox regression model to correlate overall survival (OS) with NKG2DL RNA expression (“low” and “high” groups were created using the median expression as the cut-off point) or clinical–pathological factors. No significant differences were found in mOS based on levels of MICA (*p* = 0.402), MICB (*p* = 0.202), ULBP1 (*p* = 0.081), ULBP2 (*p* = 0.215), ULBP3 (*p* = 0.647), RAET1E (*p* = 0.165), and RAET1L (*p* = 0.166). Significant differences were found in RAET1G (low vs. high; 131.5 months vs. 132.0 months; *p* = 0.044; HR = 1.4, 95%CI: 1.01–1.96; Figure 4). In relation to clinical–pathological factors, no differences were found in terms of histology (ductal vs. lobular; *p* = 0.510). Differences were found in tumor stage code (T1 vs. T2–4; *p* = 0.026), nodal stage code (N0 vs. N+; *p* < 0.001), and molecular subtype (*p* = 0.008).

The significant variables in the univariate model were incorporated into a multivariate model. The independent prognostic factors identified were molecular subtype Luminal A (*p* = 0.020, aHR = 0.58, 95%CI: 0.37–0.92) and N+ (*p* = 0.001, aHR = 1.91, 95%CI: 1.31–2.80).

**Figure 5:**
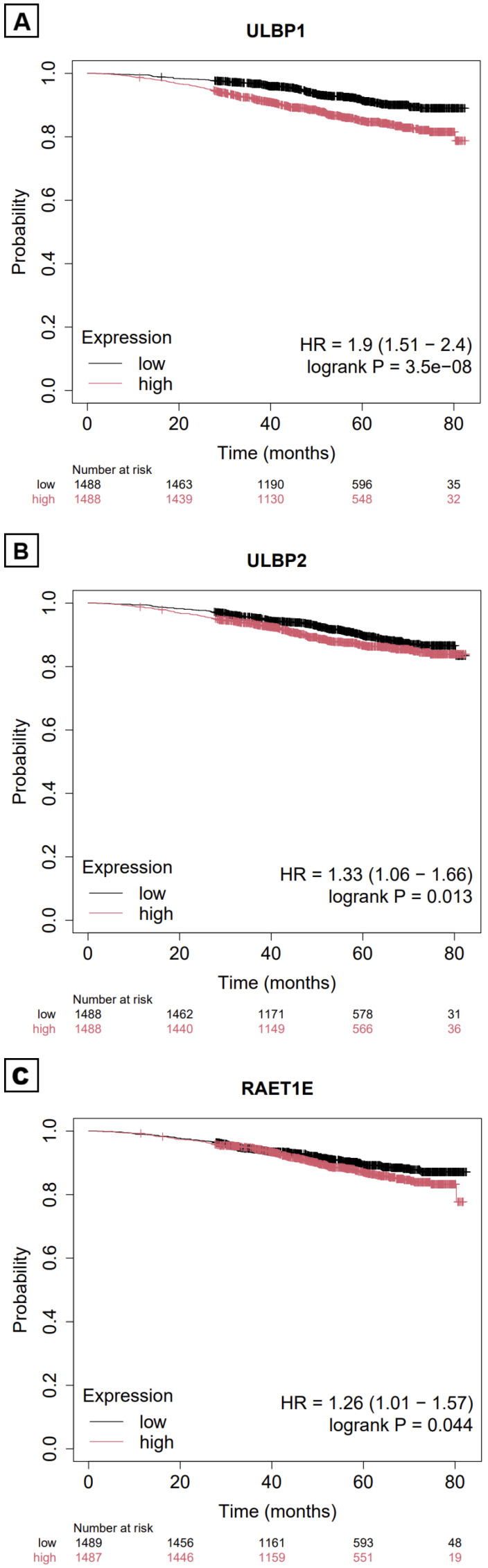
Survival curves for overall survival stratified by low–high levels of ULBP1 (**A**), ULBP2 (**B**) and RAET1E (**C**). Obtained from https://www.kmplot.com/analysis/ (accessed on 23 December 2025). Parameters: mRNA > RNA-seq > breast cancer. Patients were split by median. Survival: OS (*n* = 2976).

A similar analysis was performed using another database, The Kaplan–Meier (KM) Plotter Online Tool [https://www.kmplot.com/analysis/ (accessed on 23 December 2025)]. It has RNA-seq data on 2976 breast cancer patients obtained from the GEO and EGA repositories [27]. It allowed us to analyze the relationship between the relevance of different NKG2DL RNA expressions (“low” and “high” groups were created using the median expression as the cut-off point) and patients’ clinical outcomes (OS). No significant differences were found in mOS when stratified by low–high levels of MICA (*p* = 0.48), MICB (*p* = 0.70), ULBP3 (*p* = 0.62), RAET1G (*p* = 0.29) and RAET1L (*p* = 0.75). Significant differences were found in ULBP1 (*p* = 0.001; HR = 1.9, 95%CI: 1.5–2.4), ULBP2 (*p* = 0.013; HR = 1.3, 95%CI: 1.1–1.7) and RAET1E (*p* = 0.044; HR = 1.26, 95%CI: 1.01–1.6) (Figure 5). When specifically analyzing the TNBC subtype (*n* = 126) and the basal subtype (*n* = 309), no significant differences in mOS were found based on NKG2DL expression.

## 3. Narrative Review

We conducted a keyword-search-based literature review using PubMed. The following output was introduced in December 2025: (“breast neoplasms”[MeSH Terms] OR BREAST CANCER[Text Word] AND NKG2D) OR (“breast neoplasms”[MeSH Terms] OR BREAST CANCER[Text Word] AND NKG2DL). A total of 114 studies were obtained and their distribution over time was recorded (Table S5).

### 3.1. Potential Resistance Mechanisms

The mechanisms by which each cancer (not only BC) can evade the NKG2D system are numerous and complex. Table 4 summarizes the available evidence.

### 3.2. Findings in Breast Cancer Patients

Over the past 20 years, data on NKG2DL in patients with BC has been collected. Table 5 summarizes the available evidence.

### 3.3. Activating Mechanisms

Various mechanisms are being studied to improve the function of the NKG2D system. Table 6 shows the available evidence.

## 4. Discussion

In this study, we reviewed the published evidence and in silico data available on NKG2DL in BC. At the transcriptional level, NKG2DL is expressed in BC cell lines and tumor samples. This expression is robust compared to other tumor types. However, expression levels are lower than those of some genes whose targets are currently used in the treatment of metastatic BC, such as TACSTD2 or ERBB2. Nevertheless, we have the case of CD274 (which also participates in the immune response) that resembles that of NKG2DL. It should be noted, however, that in metastatic TNBC, immune checkpoint inhibitors only work in a selected PD-L1-positive population [5], so we wonder whether future therapies against the NKG2D/NKG2DL axis will also need to be selected based on expression. One way to stratify indirectly is to see which subpopulations have higher levels of NKG2DL. Using TCGA data, we see that expression is generally higher in ductal histology, T ≥ 2, and basal molecular subtype. This last molecular aspect could support the idea that the NKG2D/NKG2DL axis could be a target in TNBC.

An important aspect is that in silico studies of healthy breast tissue also show NKG2DL mRNA expression. This could lead to problems in terms of “on target, off tumor” toxicities. However, the expression of RNA transcripts does not necessarily imply effective protein expression. There is evidence that healthy tissues can inhibit NKG2DL protein expression despite mRNA transcripts existence through post-transcriptional mechanisms (e.g., miRNA) or post-translational mechanisms (e.g., preventing protein translocation from the endoplasmic reticulum to the cell surface) [133]. However, the in silico data we have on protein expression to clarify this is scarce. Therefore, to determine the evidence of NKG2DL protein expression in BC, we conducted a narrative review in PubMed. As previously mentioned, there is NKG2DL protein expression in BC tumor cells, while immunohistochemical staining in non-tumor border tissues is low. This supports the use of NKG2DL as a tumor target.

**Figure 6:**
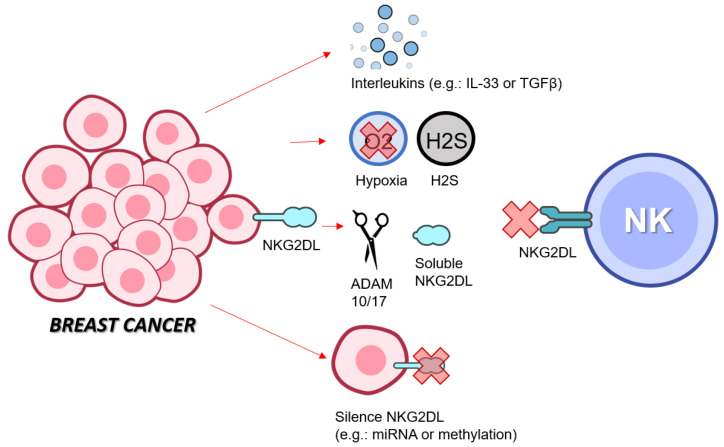
Potential mechanisms by which BC tumor cells can evade NK cell cytotoxicity mediated by NKG2D/NKG2DL. Illustrations obtained from https://bioart.niaid.nih.gov/ (accessed on 5 February 2026) [137].

There is evidence that NKG2DL protein expression may be associated with aggressiveness (higher TNM stage, higher histological grade, etc.) [64]. In fact, this is what we have seen at the transcription level with TCGA data. However, this also seems to be accompanied by greater dysfunction of NKG2D+ effector cells. This is where the resistance mechanisms described above come into play and form part of the complex process of immune editing that occurs in the tumor to evade the immune system. Among these, the secretion of soluble factors appears to be important. It has been observed that soluble NKG2DL levels are increased in patients with BC and that these levels are associated with aggressiveness [30,78]. In the proposed model, more aggressive tumors would produce more NKG2DL transcripts, but part of the translated proteins would be shed into the microenvironment and bloodstream. This shedding, among other mechanisms, would cause dysfunction of NKG2D+ effector cells (Figure 6).

The data on prognostic value are mixed. With TCGA data, only high RAET1G expression is associated with a worse prognosis. However, the absolute difference between the two subgroups is not clinically significant (0.5 months), with a modest HR (HR 1.4) and no significance in the multivariate analysis. With KMPlot data, high expression of ULBP1, ULBP2, and RAET1E is associated with a worse prognosis. The study by Madjd et al. finds that high expression of MICA/MICB and ULBP-2 is associated with a worse prognosis [63]. However, this is not unique to BC. Similar studies in colorectal or ovarian cancer have identified specific NKG2DLs as prognostic factors [134,135]. One challenge in analyzing NKG2DL expression is that at least eight different ligands are being evaluated, which may lead to concordance issues across datasets. In addition, the distribution of patients into “low” and “high” expression subgroups depends on the cut-off values chosen, influencing subgroup size and survival analyses. In future studies, it may be valuable to develop a composite score integrating expression across multiple NKG2DLs to provide a more robust and reproducible assessment of their prognostic relevance.

Finally, we reviewed potential therapeutic strategies currently being developed to target the NKG2D/NKG2DL axis in BC. On the one hand, it is interesting to note how classic chemotherapy or monoclonal antibodies used in routine clinical practice are capable of overcoming tumor immune-editing mechanisms and promoting NK-mediated cytotoxicity. Looking ahead, clinical studies in humans appear to be focused on adoptive T-cell therapy (especially CAR technology). A search on www.clinicaltrials.gov (accessed on 27 December 2025) using NKG2D as the “intervention/treatment” yields 44 studies. In 41 studies, the target population is cancer patients (12 studies may have included breast cancer patients). Most studies are phase I (with some phase I/II). The experimental treatment is adoptive cell therapy in 39 studies (35 of which are CAR-based) [136].

## 5. Future Directions

**Figure 7:**
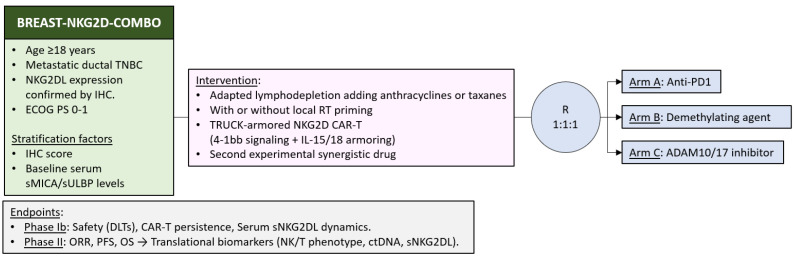
Potential phase Ib/II clinical trial combining CAR-T therapy with other modalities in metastatic TNBC NKG2DL+. ECOG PS = European Eastern Cooperative Oncology Group performance status, DLTs = dose-limiting toxicities, ctDNA = circulating tumor DNA.

However, as we have seen, the tumor develops different strategies to evade NKG2D+ cells. Therefore, it seems necessary to develop new approaches to overcome the resistance in the NKG2D/NKG2DL axis [138]. One option would be to combine adoptive cell therapy with other treatment modalities (e.g., concomitant/sequential use of chemotherapy, immune checkpoint inhibitors, ADAM inhibitors, deacetylating agents, etc.). Another option is to optimize adoptive cell therapy with technologies such as CIK, CAR-NK or TRUCK (T cells redirected for antigen-unrestricted cytokine-initiated killing) [139]. In Figure 7, we propose a potential phase Ib/II clinical trial that could be considered after positive preclinical studies have been conducted.

An important consideration is CAR-T cell dosing. Based on the CAR4SAR study in sarcoma, weekly intravenous doses of 3 × 10^6^ cells/kg (up to three doses, with discontinuation in case of ≥grade 3 toxicity) are proposed as a rational reference framework [140].

Anthracyclines and taxanes are two chemotherapy drugs widely used in BC, and there is also evidence that they increase NKG2DL expression [85,86]. However, the classic conditioning regimen for CAR-T therapy uses other chemotherapy modalities (cyclophosphamide and fludarabine) and does not use anthracyclines or taxanes. In fact, the concomitant use of anthracyclines with CAR-T therapy is not recommended [141]. Therefore, the question arises as to how these cytostatic drugs could be incorporated (added to the lymphodepletion regimen or as bridge therapy prior to CAR-T infusion). The potential toxicity associated with the addition of these cytostatic agents must be carefully considered. Importantly, it remains to be determined whether lower, subtherapeutic doses sufficient to induce NKG2DL expression could be employed, potentially limiting treatment-related toxicity.

Another drug that is effective in TNBC is pembrolizumab (anti-PD1) [5]. A study has previously been mentioned in which the administration of anti-PD1 could increase NKG2D+ effector cells [42]. Another interesting target is the methylation of NKG2DL genes. This methylation appears to be described as one of those responsible for immune evasion in acute myeloid leukemia and agents like 5-aza-cytidine could reverse this epigenetic silencing [58]. Old drugs with newly discovered functions are also being studied, such as spironolactone, a diuretic that could increase the expression of NKG2DL in tumor cells [142]. Other drugs of interest currently in preclinical research are ADAM10/17 inhibitors and monoclonal antibodies against sNKG2DL [138].

## 6. Conclusions

In summary, available in silico data and published evidence indicate that NKG2DL ligands are transcriptionally expressed in BC across cell lines and tumor samples, with higher levels generally observed in biologically aggressive subtypes (especially TNBC). The frequent coexistence of NKG2DL expression with impaired NKG2D-positive effector cell function, together with multiple tumor-driven resistance mechanisms—particularly ligand shedding—highlights the complexity of immune surveillance in BC. However, this raises a starting point for the development of new therapies addressing this NKG2D/NKG2DL axis (with adoptive cell therapy taking on particular importance).

## Figures and Tables

**Table 1:** RNA expression in TPM (mean ± SD) in different immortalized human cell lines. BC = breast cancer (*n* = 29). LL = leukemia/lymphoma (*n* = 101). NB = neuroblastoma (*n* = 18). RB = retinoblastoma (*n* = 9). Data obtained from https://celldive.dsmz.de/ (accessed on 23 December 2025).

Gene (TPM)—Mean ± SD	BC	LL	NB	RB
MICA	26.1 ± 20.7	9.8 ± 6.4	2.9 ± 8.5	1.1 ± 2.7
MICB	11.0 ± 13.5	19.0 ± 13.7	0.6 ± 1.3	3.0 ± 5.7
ULBP1	2.1 ± 2.2	1.7 ± 3.6	1.3 ± 2.0	0.2 ± 0.3
ULBP2	6.8 ± 6.7	2.0 ± 4.3	1.5 ± 1.8	4.2 ± 3.9
ULBP3	3.6 ± 3.9	1.1 ± 1.9	1.0 ± 1.3	0.8 ± 0.8
RAET1E	1.5 ± 2.2	0.9 ± 3.8	1.4 ± 5.7	0.0 ± 0.0
RAET1G	1.4 ± 3.0	0.1 ± 0.3	4.1 ± 16.8	0.0 ± 0.0
RAET1L	1.4 ± 4.5	0.2 ± 1.6	0.0 ± 0.0	0.0 ± 0.0
CD274	5.6 ± 11.6			
TACSTD2	220.1 ± 213.0			
NECTIN4	21.8 ± 27.6			
ERBB2	408.2 ± 783.5			

**Table 2:** RNA expression in nTPM (mean ± SD) in healthy breast tissue. Data obtained from https://www.proteinatlas.org/ and http://www.gtexportal.org/home/ (both accessed on 23 December 2025).

Gene (nTPM)—Mean ± SD	Breast
MICA	29.1 ± 10.6
MICB	2.1 ± 1.2
ULBP1	0.0 ± 0.1
ULBP2	0.7 ± 0.8
ULBP3	1.5 ± 2.0
RAET1E	0.4 ± 0.3
RAET1G	0.5 ± 0.4
RAET1L	0.0 ± 0.1

**Table 3:** Baseline characteristics of selected TCGA BC patients. Data obtained from https://www.proteinatlas.org/ and http://www.cbioportal.org/ (both accessed on 23 December 2025).

Baseline Characteristics	N = 1009
Age (years)—median (range)	58 (26–90)
Female sex—n (%)	1009 (100%)
Histology—n (%)	
- Ductal	721 (71.5%)
- Lobular	188 (18.6%)
- Mixed	28 (2.8%)
- Mucinous	17 (1.7%)
- Other	41 (4.1%)
- Unknown	14 (1.4%)
Molecular subtype (PAM50)—n (%)	
- Luminal A	473 (46.9%)
- Luminal B	180 (17.8%)
- HER2-enriched	72 (7.1%)
- Basal	162 (16.1%)
- Normal	34 (3.4%)
- Unknown	90 (8.9%)
Tumor stage code—n (%)	
- T1	263 (26.1%)
- T2	584 (57.9%)
- T3	124 (12.3%)
- T4	35 (3.5%)
Nodal stage code—n (%)	
- N0	475 (47.1%)
- N1	335 (33.2%)
- N2	114 (11.3%)
- N3	69 (6.8%)
RNA expression—mean ± SD (pTPM)	
- MICA	4.4 ± 6.3
- MICB	1.1 ± 1.9
- ULBP1	0.9 ± 1.7
- ULBP2	2.0 ± 3.6
- ULBP3	1.5 ± 2.1
- RAET1E	1.6 ± 3.3
- RAET1G	0.7 ± 1.1
- RAET1L	1.0 ± 3.1
- CD274	2.2 ± 2.6
- TACSTD2	292.2 ± 213.4
- NECTIN4	37.7 ± 27.2
- ERBB2	319.6 ± 680.9

**Table 4:** Evidence of resistance mechanisms.

Resistance Mechanisms	Evidence
Soluble forms of NKG2DL (They can block NKG2D+ lymphocytes, preventing their action in the tumor microenvironment and facilitating immune evasion)	- Exosomes released by BC cell lines express NKG2DLs and reduce the proportion of NKG2D+ lymphocytes [28,29]. - NK cells incubated in soluble MICA (sMICA) demonstrate reduced cytotoxicity [30]. - The “a disintegrin and metalloproteases” (ADAMs) 10 and 17 are largely responsible for the generation of sMICA-MICB [31]. - Senescent cancer cells (SCCs) treated with antineoplastic agents upregulate NKG2DLs but also metalloproteases (MMPs), which causes their shedding [32].
Anesthetics	- Serum from patients undergoing breast tumorectomy anesthetized with sevoflurane–opioid (versus paravertebral block and propofol) was associated with NK cell dysfunction. However, it was not associated with a deficit in NKG2D [33]. - Sevoflurane attenuates NK cell-mediated cancer cell lysis. It downregulates the mRNA/protein expression of the NKG2DLs in cancer cell lines without affecting MMP or soluble NKG2D levels [34]. - Propofol modifies NKG2DL levels (increasing them in some cell lines and decreasing them in others) without altering MMP levels [35].
Metabolic syndrome	- Streptozotocin-induced diabetes accelerates tumor growth, which is associated with decreased NK cell cytotoxicity (reduced NKG2D) [36]. - LDL cholesterol inhibits NKG2D and the cytotoxicity of phosphoantigen-expanded Vγ9Vδ2 T cells [37]. - A mouse model of obese postmenopausal breast cancer shows increased tumor growth and decreased levels of circulating NK cells with increased expression of NKG2DLs in adipose tissue [38].
Hypoxia	- Hypoxia did not alter the expression of MICA/MICB but did increase their extracellular shedding, potentially explaining resistance to γδTc cytotoxicity [39]. - Extracellular lactate flux and acidified tumor microenvironment may disrupt NK cell function [40].
H_2_S (synthesized by enzymes cystathionine β-synthase (CBS) and cystathionine γ-lyase (CSE))	- Silencing CBS/CBE increases MICA and ULBP2 RNA transcripts [41]. - Ectopic expression of miR-939-5p represses CBS/CSE transcript and protein levels, diminishes H_2_S production, upregulates NKG2DLs and attenuates triple-negative hallmarks [42].
IL-33	- Especially in the presence of ST2/IL1R-L1, promotes a reduction in intratumoral NKG2D+ cells and favors tumor progression [43]. - Blocking PD-1 with an antibody in ST2 knockout mice increases NKG2D+ T cells in the tumor microenvironment [44].
P53	- Murine p53 missense mutation G242A (corresponding to human G245A) suppresses the activation of host NK cells. p53 can modulate expression by cancer cells of Mult-1 (murine NKG2DL) and H60a [45].
PI3K/AKT/GSK-3β	- Inactivated GSK-3β downregulates the expression of NKG2DLs via ROS and eIF2B [46].
EHMT2	- EHMT2 loss increases AZGP1 and decreases TGF-β1 levels, resulting in elevation of NKG2DLs (MICB and ULBP3), chemokines in cancer cells, and the paracrine stimulation of NK cell function [47].
ATM	- Associated with MICA upregulation. In triple-negative breast cancer, there is ATM downregulation, which may influence its immunoreactive profile [48].
Heat shock factor 1 (HSF1)	- HSF1 upregulates MICA/MICB expression. NZ28 completely inhibits HSF1 blocking the MICA/MICB membrane expression on tumor cells and thereby strongly inhibits NK cell-mediated cytotoxicity [49].
Podocalyxin-like protein 1 (PCLP1)	- CD34-related sialomucin. Overexpressed in cancer cells. Associated with decreased NKG2D levels in NK cells [50].
IGFBP-3	- May promote breast tumor growth through T-cell dysfunction, but does not alter NKG2D levels [51].
Breast cancer stem-like cells (BCSCs)	- Aberrantly express oncogenic miR20a, which inhibits MICA/MICB, protecting them from NK cell-mediated cytotoxicity [52]. - Activate platelets inducing TGFbeta production and inhibiting NK cells (reducing NKG2D expression) [53].
Adipose-derived stem cells (ASCs)	- ASCs from breast tumors exert an immunosuppressive effect on NK cells, showing reduced NKG2D levels [54]. - MICB expression in ASCs from healthy subjects and those with stage II breast cancer is higher than in those with stage III cancer. However, this is reversed with the administration of INFγ [55].
“Central” markers of dormancy/quiescence	- Breast cancer brain metastasis cells NKG2DL+ express H2BK, IGFBP5 and EphA5 [56].
Ectopic expression of NKG2D	- Induced expression of NKG2D in cancer cell lines may paradoxically have a pro-tumor effect [57].
DNA methylation	- Can contribute to the absence of NKG2DL expression during tumor progression [58].
RNA	- miR-519a-3p interferes with tumor-cell-killing NK cells via downregulation of MICA and ULBP2 [59]. - UCA1 (an lncRNA) is upregulated in NK-resistant cell lines. UCA1 upregulates ULBP2 via the transcription factor CREB1, while it upregulates ADAM17 by “sponging” the miR-26b-5p. ADAM17 facilitates the shedding of soluble ULBP2 [60].
Xanthine oxidoreductase (XOR)	- Inhibition of XOR blocks the expression of MICA/MICB [61,62].

**Table 5:** Available evidence for NKG2DL in BC.

NKG2DL in BC	Evidence
NKG2DL is expressed in BC. NKG2DL expression is low in surrounding non-tumor tissues. NKG2DL expression could be associated with a worse prognosis.	- Madjd et al. studied 530 BC patients. MICA staining was detected in 97% of blocks. It was not detected in the extracellular stroma but was detected in adjacent normal epithelial cells, endothelial cells, and some tumor-infiltrating leukocytes. MICA expression was associated with worse grade and nodal stage indicating a worse prognosis [63]. - Shen et al. studied 92 BC patients. 92.2% of the samples tested positive for MICA/MICB by immunohistochemistry (compared to only 15.6% of healthy tissues). MICA/MICB expression was inversely correlated with TNM staging (this was not detected with ULBP1, ULBP2, and ULBP3) [64]. - de Kruijf et al. studied 677 primary BC tumors. They detected NKG2DL expression (MICA/MICB 50%, ULBP1 90%, ULBP2 99%, ULBP3 100%, ULBP4 26% and ULBP5 90%). Low levels of MICA/MICB and ULBP-2 were associated with a longer relapse-free period (RFP) [65]. - BC cells show upregulated NKG2DLs compared with primary breast epithelial cells. NKG2DLs activate mucosal-associated invariant T innate cells [66].
However, there is a dysfunction in NKG2DL expression and NKG2D+ cell function in BC. The recovery of NK function may be a good prognostic sign.	- Elsabbagh et al. detected higher m^6^A levels in the 3′ untranslated region (3′UTR) accompanied by a marked reduction in their corresponding mRNA levels in BC patients compared to controls [67]. - BC patients have decreased NK cell cytotoxicity and lower NKG2D expression [68]. Malignant mammary-tumor-infiltrating NK cells show reduced expression of activating receptors such as NKG2D compared to circulating NK cells of healthy donors [69]. Arianfar et al. showed that NK subtypes showed lower NKG2D expression in breast cancer (*n* = 26) patients than healthy subjects (*n* = 12) [70]. - However, Kawaguchi et al. detected no differences in NKG2D levels between patients with primary tumors (*n* = 12), metastases (*n* = 30), and healthy subjects (*n* = 6) [71]. Darvishvand et al. analyzed 30 patients with breast cancer and 20 healthy subjects. Around 15% of lymphocytes in peripheral blood had NK cell characteristics. No differences were found between the two groups [72]. - Wu et al. detected Vδ1^+^ T cells (which could be activated innately via the NKG2D receptor) in human BC tumors. When paired tumor and nonmalignant samples from 11 patients with TNBC were analyzed, PFS (progression-free survival) and OS correlated with Vδ1^+^ cell representation [73]. - In TNBC, there are more NKG2D+ cells in tumors without nodal involvement (N0) [74]. - Verma et al. showed that, with regard to controls (*n* = 10), patients with locoregional BC (*n* = 25) had a lower absolute number of NK cells and lower cytotoxicity. This decrease was corrected in patients who had a complete pathological response with neoadjuvant chemotherapy and/or after surgery [75]. - Lobo-Martins et al. studied 44 BC patients treated with hormone therapy and cyclin inhibitors. The treatment decreased Vδ2+ T cells expressing NKG2D at 3 months. There was an increase in CD8+ T cells expressing NKG2D in responders (PFS > 6 months) [76].
There is expression of soluble forms of NKG2DL in BC. sNKG2DL could also be associated with a worse prognosis.	- sMICA/sMICB levels are higher in breast cancer patients than in healthy individuals [77]. sMICA levels correlate with tumor staging [30,78]. This is also detected in saliva and urine [79]. - Seller et al. collected serum from 140 BC patients, 20 ductal carcinoma in situ patients and 20 healthy volunteers. Higher sNKG2DL serum levels were associated with TNM stage and grading. Low sMICA serum levels were associated with significantly longer PFS and OS [80].
Germinal alterations in NKG2DL could predispose to BC	- Lavado-Valenzuela et al. studied 110 BC cases and 121 controls in Spain. They found that the MICA-A5 allele appears less frequently in BC, while the MICA-A5.1 allele appears more frequently in HLA-B7-positive BC [81]. - Ouni et al. detected that MICA-129 Met/Val polymorphism is associated with higher breast cancer risk in Tunisian women <40 years old [82]. Ouni et al. also showed that MICA-129 Met/Val polymorphism is associated with BC risk in Tunisian women [83].

**Table 6:** Evidence of activating mechanisms.

Activating Mechanisms	Evidence
Chemotherapy	- *Arsenic trioxide* increases NKG2DL expression (especially ULBP1), promoting cytotoxicity [84]. - *Docetaxel* promotes NKG2D expression and enhances trastuzumab-mediated ADCC [85]. - *Gemcitabine* induces MAP kinase activation and MICA/B expression [62]. - *Epirubicin* increases NKG2DL expression (MICA, ULBP1, and ULBP2) in tumor cells and promotes NK cell-mediated cytotoxicity [86]. - *Doxorubicin* plus TAK-981 (SUMOylation inhibitor) downregulates ULBP2 through suppression of the NF-κB pathway inhibiting tumor growth and enhancing NK cell activity [87].
Cytokines	- *IL-15* upregulates NKG2D expression in NK cells previously inhibited by sMICA and increases NK cell cytotoxicity toward breast cancer cells [30]. IL-15 increases NKG2D+ cell levels in BC metastases (the addition of everolimus has no synergistic effect) [88]. - *IL-2 and IL-15* lead to greater sensitivity to cytotoxicity mediated by NK cells to BSCS (which express higher levels of MICA, ULBP1, and ULBP2) [89]. - *Bryostatin 1 and ionomycin combined with IL-2, IL-7, and IL-15* can reactivate peripheral blood mononuclear cells (including NK cells) of patients with BC inhibited by myeloid-derived suppressor cells [90]. - *IFNγ and TNFα* enhanced NK cell infiltration with increased expression of activating receptors (e.g., NKG2D) in BC spheroids. However, regardless of treatment, markers of NK cell exhaustion, such as PD-1 and CTLA-4, were also upregulated [91].
Antibodies	- *Anti-TGFβ* increases NKG2D/NKG2DL expression, promoting tumor lysis [92]. - *Anti-EGFR* (cetuximab) promotes antibody-dependent cellular cytotoxicity (ADCC), especially when administered with IL15 (which increases NKG2D expression) [93]. - *Anti-CD6* (UMCD6) augments killing of breast cancer cells through direct effects on both CD8+ T cells and NK cells (upregulating NKG2D) [94]. - *Anti-CD6* (itolizumab) increases the cytotoxicity of CD8 T and NK cells over CD318+ tumor lines, reverses the NKG2A/NKG2D ratio, and increases granzyme B and IFNγ production [95]. - *Anti-CD137* agonist (urelumab) can overcome TGFβ-mediated inhibition of human NK cell proliferation, NKG2D expression and antitumor function [96].
Fusion proteins	- *Anti-HER2:IgG3-Rae-1β* (murine NKG2DL) promotes cytotoxicity [97]. - *Anti-NKG2D:IgG1 with amino acid exchange S239D/I332E in their Fc part* improves degranulation, ADCC, and IFN-γ production of NK cells in response to breast cancer cells. In addition, it increases sensitivity to trastuzumab in HER2-low targets [98]. - *NKG2D:anti-HER2 acts synergistically with B7-H6(NKp30):anti-HER2 and AICL(NKp80):anti-HER2* to produce ADCC [99]. - *mAb04(anti-VEGF):MICA* demonstrate superior antitumor efficacy compared to a combination therapy of mAb04 + docetaxel or bevacizumab + docetaxel [100]. - *IL-15SA:IL-15RαSu* enhances subpopulations of NK and has an antitumor effect [101]. - *ULBP2:anti-HER2* triggers NK cell-mediated killing of HER2-positive BC cells [102]. - *NKG2D:CD3 and NKG2D:CD16* efficiently activate both NK and T cells against TNBC [103]. - *NKG2D:anti-HER2* enhances NK cell activation and cytokine production [104] - *Anti-Fzd7:MICA* significantly enhances the cytotoxicity of NK cells against hypoxic TNBC cells [105].
Adoptive T-cell therapy	- *CAR anti-NKG2D* exhibits antitumor activity against TNBC cell lines ex vivo and in vivo in a mouse model [106]. - *Cytokine-induced killer (CIK)* cells in combination with cetuximab showed an antitumor effect in TNBC lines ex vivo and in vivo in a mouse model [107]. - *NK-92 cell line* has an antitumor effect on spheroids of cell lines [108]. - *Intratumoral injection of allogeneic NK cell*: Chemotherapy was found to upregulate the expression of NKG2D ligands at both mRNA and protein levels on cancer cells, increasing their susceptibility to NK cell-mediated cytotoxicity [109]. - *Bispecific T-cell engager CAR-T (BiTE CAR-T) cells targeting mesothelin (MSLN) and secreting NKG2D-Bispecific T*-*cell Engagers (BiTEs) to engage NKG2DL*: BiTE CAR-T cells exhibit superior cytotoxicity, T-cell activation, and cytokine production against heterogeneous target cells compared to MSLN CAR-T [110]. Their use is being studied with closed-loop sonothermogenetics for spatial and temporal control of CAR-T cells [111].
Other types of immunotherapy	- *Adenovirus E1A*: Infection of cancerous cell lines increases NKG2DL expression and facilitates cell lysis [112]. - *Antitumor vaccines*: Inclusion of NKG2DLs as adjuvants is being evaluated [113].
Other compounds	- *Resveratrol* upregulates the protein and mRNA expression of MICA and MICB [114]. Resveratrol potentiates ULBP2-mediated immune eradication of BC cells by NK cells through the downregulation of miR-17-5p and concurrent activation of the MINK1/JNK/c-Jun cascade [115]. Nevertheless, ectopic expression of miR-17-5p represses oncogenic mediator STAT3 and oncogenic lncRNA H19, inducing ULBP2 expression [116]. - *Inhibition of MCT4* (monocarboxylate transporter-4) upregulates NKG2D/NKG2DL, enhancing the cytotoxicity of NK cells [40]. - *P. ostreatus* polysaccharides induced NK cell cytotoxic effects against BC (correlated with NKG2D upregulation). These cytotoxic effects were enhanced in the presence of IL-2 [117]. - *3′-O-acetylvitexin* (3OA) produced a dose-dependent repression of TNBC viability, colonogenicity and migration capacity. It produced a marked dose-dependent repression of miR-20a with a concomitant dose-dependent increase in MICA/MICB expression [118]. - *Tanshinol* can interfere with the negative regulation of NK cell functions by TGF-β1, mainly through promoting the expression of NKG2D and its molecular chaperone DAP10 [119]. - *Amygdalin–folic acid nanoparticles*, as radiosensitizers, interfere with TGFβ, INF**γ**, IL2, IL6 and VEGF, upregulating NKG2D [120]. - *Ginsenoside Rh2* retards the growth and metastasis of BC through boosting the cytotoxic function of NK cells. It prevents the formation of sMICA and upregulates MICA in vitro and in vivo [121]. - *Cordycepin and C. militaris ethanolic extract* increases NKG2D/NKG2DL and elevated cancer cell death [122]. - *Dichloroacetate* significantly increases the mRNA of MICA/B and ULBP1 exclusively in cells that express normal p53 [123].
Bisphosphonates	- Promote cell lysis mediated by Vγ9Vδ2 T cells via γδ TCR, perforin–granzyme pathway, and NKG2D (although they do not modify MICA expression) [124]. - Zolendronic acid increases NKG2D in peripheral blood mononuclear cells from BC patients [125].
Demethylating agents	- *5-azacytidine and 5-aza-2’-deoxycytidine* can restore the expression of NKG2DL [58].
HER2/HER3 pathway	- Promotes NKG2D-MICA/B recognition by NK cells and T cells [126].
Nanotechnology	- *Biopolymer* implants can enhance the efficacy of adoptive NK-CAR cell therapy [127]. - *Nanoemulsion system* to co-deliver TGF-β inhibitor and selenocysteine enhances the lytic potency of NK92 cells (via enhanced NKG2DL expression on tumor cells and stimulated NKG2D expression on NK92 cell) [128]. - *Selenadiazole-derivative-loaded metal azolate framework* (PSeD@MAF-4(R)) exhibits synergistic effects with NK cells in inhibiting tumor cell growth by upregulating NKG2D/NKG2DL [129]. - *Ruthenium polypyridyl complex pretreatment* together with NK cells can maximize the interactions between NK and tumor cells via upregulating NKG2D and its multiple ligands [100]. - *Nanoparticle@K3-E3@V_H_Hs* (nanoparticle platforms in which nanobodies are non-covalently displayed on the surface of nanoparticles through a specific heterotetrameric peptide assembly K3–E3) can target multiple cell surface receptors (e.g., NKG2DL) [130].
Exercise	- *Acute exercise* does not alter NKG2D levels. However, different *chronic exercise* techniques (standard endurance training vs. polarized endurance training after a cardiopulmonary exercise test) do modify it [131].
Radiation	- *Low doses of chronic internal radiation* in mice produce a decrease in NKG2D expression and an increase in NKG2DL expression at 3.5 months, which subsequently normalizes [132].

**Figure A5:**
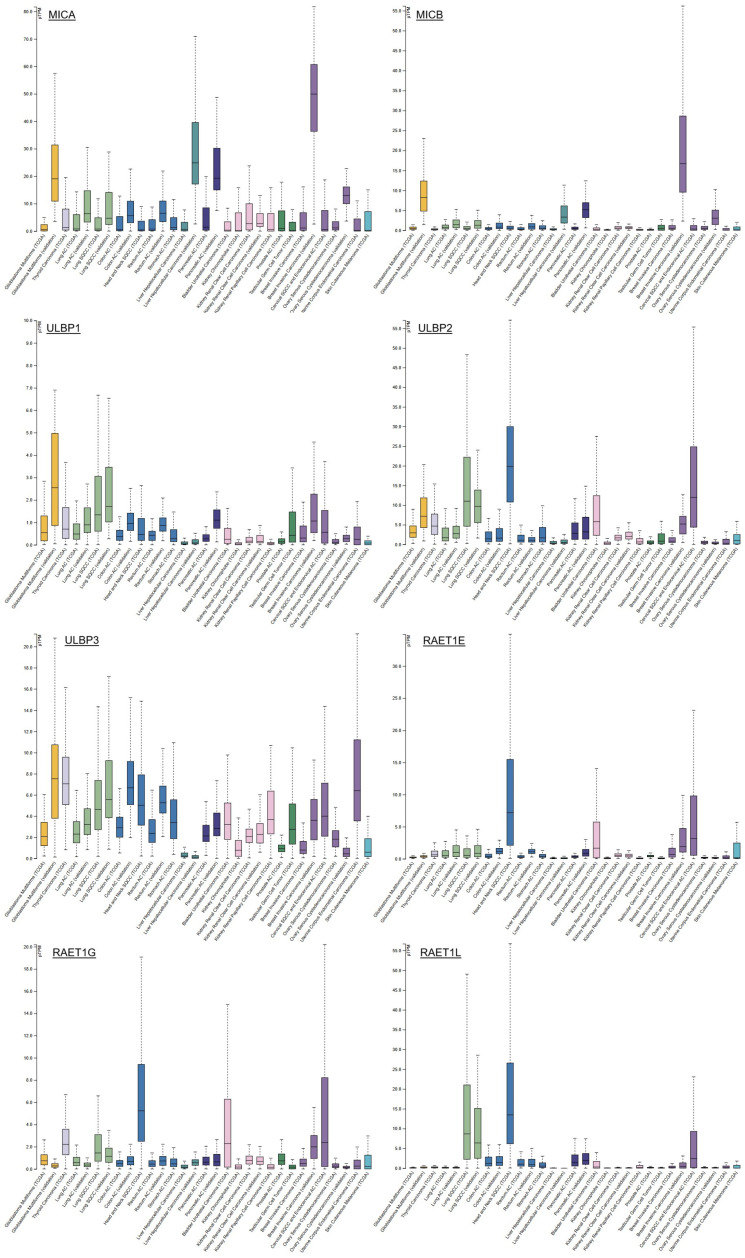
RNA expression in pTPM of NKG2DL in different tumors. Obtained from https://www.proteinatlas.org/ (accessed on 20 January 2026) [143,144,145,146,147,148,149,150].

## Data Availability

The public databases listed in the review (DSMZCellDive, ShinyTHOR, GTEx, The Human Protein Atlas, cBioportal, KMPlot, etc.) were used. They allow free use for academic research, following the guidelines of the authors of each database and with proper citation. The Excel tables created from this data are included in the Supplementary Materials section. Specific questions may be directed upon reasonable request to the corresponding author.

## Supplementary Materials

The following supporting information can be downloaded at https://www.mdpi.com/article/10.3390/ijms27041848/s1.

## Abbreviations

The following abbreviations are used in this manuscript:

3′UTR	3′ untranslated region
95%CI	95% confidence interval
ADAM	A disintegrin and metalloprotease
ADC	Antibody–drug conjugate
aHR	Adjusted hazard ratio
ASC	Adipose-derived stem cell
ATM	Ataxia-telangiectasia mutated
BC	Breast cancer
BCSC	Breast cancer stem-like cells
BiTE	Bispecific T-cell engager
CAR	Chimeric antigen receptor
CBS	Cystathionine β-synthase
CIK	Cytokine-induced killer
CSE	Cystathionine γ-lyase
ctDNA	Circulating tumor DNA
CTLA4	Cytotoxic T-lymphocyte-associated protein 4
DLTs	Dose-limiting toxicities
ECOG PS	European Eastern Cooperative Oncology Group performance status
EGFR	Epidermal growth factor receptor
EHMT2	Euchromatic histone-lysine N-methyltransferase 2
EphA5	Ephrin type-A receptor 5
ER	Estrogen receptor
GSK-3β	Glycogen synthase kinase-3 beta
H2BK	H2B.K variant histone
H2S	Hydrogen sulfide
HER2	Human epidermal growth factor receptor 2
HR	Hazard ratio
HSF1	Heat shock factor 1
IGFBP	Insulin-like growth factor binding protein
IHC	Immunohistochemistry
IL	Interleukin
INFγ	Interferon gamma
lncRNA	Long non-coding RNA
MCT4	Monocarboxylate transporter-4
Met	Methionine
MICA	Major histocompatibility complex class I-related protein A
MICB	Major histocompatibility complex class I-related protein B
miR	Micro RNA
MMP	Metalloprotease
mOS	Median overall survival
mPFS	Median progression-free survival
NK	Natural killer
NKG2D	Natural Killer group 2 member D receptor
NKG2DL	NKG2D ligand
nTPM	Normalized transcripts per million
ORR	Overall response rate
OS	Overall survival
PAM50	Predictor analysis of microarray 50
PCLP1	Podocalyxin-like protein 1
PFS	Progression-free survival
PD-L1	Protein death ligand 1
PI3K	Phosphoinositide 3-kinase
PR	Progesterone receptor
RFS	Relapse-free survival
RNA-seq	RNA sequencing
SCC	Senescent cancer cells
sMICA	Soluble MICA
sNKG2DL	Soluble NKG2D ligand
STAT3	Signal transducer and activator of transcription 3
TCGA	The Cancer Genome Atlas
TFGβ	Transforming growth factor beta
TNFα	Tumor necrosis factor alpha
TNBC	Triple-negative breast cancer
TPM	Transcripts per million
TRUCK	T cells redirected for antigen-unrestricted cytokine-initiated killing
ULBP1	UL16 binding protein 1
ULBP2	UL16 binding protein 2
ULBP3	UL16 binding protein 3
ULBP4	UL16 binding protein 4. Also: RAET1E (Retinoic Acid Early Transcript 1E)
ULBP5	UL16 binding protein 5. Also: RAET1G (Retinoic Acid Early Transcript 1G)
ULBP6	UL16 binding protein 6. Also: RAET1L (Retinoic Acid Early Transcript 1L)
Val	Valine
XOR	Xanthine oxidoreductase
